# Probing Reversible Guest Binding with Hyperpolarized ^129^Xe-NMR: Characteristics and Applications for Cucurbit[*n*]urils

**DOI:** 10.3390/molecules25040957

**Published:** 2020-02-20

**Authors:** Jabadurai Jayapaul, Leif Schröder

**Affiliations:** Molecular Imaging, Leibniz-Forschungsinstitut für Molekulare Pharmakologie (FMP), 13125 Berlin, Germany; jayapaul@fmp-berlin.de

**Keywords:** cucurbit[*n*]uril, Xe-NMR, HyperCEST, displacement assays, supramolecular systems

## Abstract

Cucurbit[*n*]urils (CB[*n*]s) are a family of macrocyclic host molecules that find various applications in drug delivery, molecular switching, and dye displacement assays. The CB[*n*]s with *n* = 5–7 have also been studied with ^129^Xe-NMR. They bind the noble gas with a large range of exchange rates. Starting with insights from conventional direct detection of bound Xe, this review summarizes recent achievements with chemical exchange saturation transfer (CEST) detection of efficiently exchanging Xe in various CB[*n*]-based supramolecular systems. Unprecedented sensitivity has been reached by combining the CEST method with hyperpolarized Xe, the production of which is also briefly described. Applications such as displacement assays for enzyme activity detection and rotaxanes as emerging types of Xe biosensors are likewise discussed in the context of biomedical applications and pinpoint future directions for translating this field to preclinical studies.

## 1. Introduction

The self-assembly of different molecules or likewise between molecules and single atoms may occur spontaneously and lead to the formation of different supramolecular structures with useful analytical features. These structures are held together by one or more types of interactions between the assembling components and the surrounding media, e.g., Hydrogen bonding, van der Waals interactions, hydrophobic and electrostatic interactions, donor-receptor effects, π-π stacking, or metal coordination, respectively [1]. The generation of these supramolecular structures typically requires only a reduced number of synthetic steps compared to generating a structure of comparable design via covalent coupling. Additionally, shape, size, and dimensions of the supramolecules can be efficiently tuned by inducing structural changes in the building blocks and by regulating different intermolecular interactions [2]. These supramolecular architectures find applications as magnetic or optical materials, as well as in catalysis, molecular recognition, drug delivery, and in transport processes [1,3,4,5,6,7,8]. Regarding biological systems, such self-assembly can lead to the generation of multicomponent complexes with a high level of structural hierarchy for enabling mechanisms related to, e.g., cell locomotion [9], transcription [10], immune response [11] and apoptosis [12], respectively. Similarly, the disassembly plays a pivotal role in modulating and terminating the actions of these supramolecular complexes in vivo induced through different stimuli such as change in temperature, pH, length of building blocks etc. In this regard, a tight balance between assembly and disassembly processes in living organisms are regulated through multiple and interconnected mechanisms. Combining enzymatic reactions with the assembly/disassembly processes provides control over the enzyme activity, e.g., by tuning the substrate release from a supramolecular complex. As another example, enzyme-instructed self-assembly (EISA) [13] is a form of supramolecular catalysis that was developed for selective achievement of small molecules-based supramolecular assemblies [14,15,16] in situ that are utilized in potential cancer therapy [17,18] and molecular imaging [19]. The supramolecular catalysis comprising enzymes [20] or synthetic catalysts [21] are helpful in regulating the kinetics involved in the assembly/disassembly processes, thus providing a spatiotemporal control over the system of interest. 

In any case, systematic understanding and monitoring of the assembly/disassembly process is an important aspect and requires methods to interrogate supramolecular systems. Optical detection is used in many studies because the fluorescence properties can significantly change between the assembled state and the free constituents. However, it can only be followed in sufficiently translucent samples and requires (switchable) fluorescent properties of one of the constituents itself or sufficient interaction with a dye. The latter one comes with a certain molecular size and thus requires a suitable interaction site within the supramolecular system. In many cases, confined spaces are involved in such systems - either in one of the constituents (as for molecular containers that bind another unit) or in the product (as for metal-organic coordination polymers). Particularly the small cavities are challenging when searching for a reporter with sufficient access but not too tight binding that might disturb the supramolecular assembly under investigation. Here, a monoatomic probe is ideal to interrogate such systems. This can be achieved through NMR with nuclei that are sensitive to their immediate molecular environment. Moreover, NMR is the method of choice for opaque conditions. To this end, ^129^Xe is a valuable NMR probe to explore the formation and accessibility of such confined spaces for sensing the assembling status because it combines several useful features:

- Xe participates in weak, non-covalent interactions, thus it does not significantly disturb the interaction of the supramolecular constituents;
- the NMR chemical shift range of ^129^Xe is rather large and provides a sensitive measure for changes in the immediate molecular environment even without engaging in more stable (or covalent) interactions;
- the noble gas can be “hyperpolarized” (hp), i.e., its spin magnetization can be artificially enhanced for significantly improved NMR sensitivity;
- in combinations with its sufficient solubility in many solvents, including water, this enables straightforward NMR detection of this monoatomic reporter for biochemical targets and many other solution samples of interest.

Altogether, ^129^Xe-NMR can deliver important insights into the properties of various supramolecular systems.

**Figure 1:**
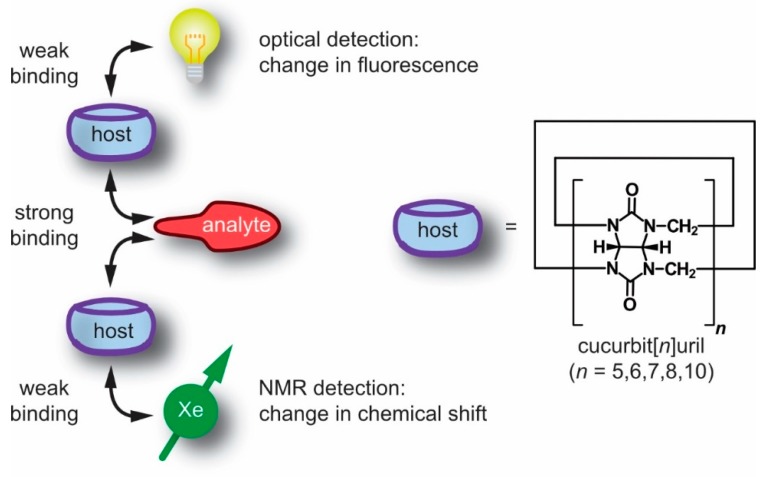
Example for extending a supramolecular-based detection assay concept from fluorescence detection to NMR readout. A fluorescent dye that binds to a molecular host is displaced upon formation of the analyte host complex. The same can be implemented with ^129^Xe atoms that bind to the cavity. In both cases, no label is directly attached to either of the supramolecular interaction partners.

Some concepts emerged from other reporter systems that are applied to supramolecular assemblies and are now available with the advantages of ultra-sensitive NMR of hp nuclei. As an example for supramolecular assays, the displacement of a dye from the dye-host pair upon the introduction of an analyte has been widely utilized to measure the absolute concentrations of the latter in so-called indicator displacement assays. Such assays highly depend upon the selectivity and sensitivity of the supramolecular host for different guests (see Figure 1). To enhance the sensitivity of such assays, a fluorescent dye with high quantum yield might be utilized in addition to sufficient high binding between the host and dye such that a large change in fluorescence intensity, i.e., quenching or enhancement, can be achieved. The host family of *p*-sulfonatocalix[*n*]arenes with lucigenin (as fluorescent guest) is an example with a high binding constant (~10^7^ M^−1^) and strong fluorescence quenching. Such indicator displacement assays have been applied to analyze several types of analytes, including citrate, glucose-6-phosphate, inositol-1,4,5-triphosphate, tartrate, malate or nitrate α-amino acids, respectively [22,23,24,25]. However, these systems suffer from a lack of specificity to target analytes similar to any other host-dye based sensing approaches [26]. 

To overcome this issue, a new method developed by Nau and co-workers, coined supramolecular tandem assays (STAs) [27,28,29,30], was utilized as a time-resolved version of indicator displacement assays involving no immediate competitor. The molecular unit displacing the reporter is generated during the course of an enzymatic reaction. Such STAs are beneficial for real-time continuous monitoring of enzymatic activity via changes in concentration of either the substrate or the product where one of them leads to competitive displacement of the dye from a macrocyclic host, e.g., cucurbit[*n*]urils (CB[*n*]s) [31]. STAs are successfully used for monitoring different enzymatic transformations linked to amino acids, biogenic amines, amino aldehydes and nucleotide phosphates, respectively [28,29,30,32]. Introduction of an enzyme in the assay leads either to an increase in fluorescence intensity with time (switch-ON assay) or a decrease (switch-OFF assay), depending upon the photophysical signature of the dye. Strong binding of either the product or the substrate to the macrocyclic host enables two different supramolecular assays, namely product- [28,31] or substrate-selective assays [30,32,33].

Extending the displacement assay concept from fluorescence to NMR is an example for emerging concepts in supramolecular systems. As such, the use of ^129^Xe also represents an implementation of the appealing concept of a “label free” approach because the reporter is not covalently attached to one of the constituents. This review summarizes several NMR techniques for studying different supramolecular assemblies with a particular focus on cucurbit[*n*]urils as hosts in hp ^129^Xe-NMR spectroscopy. The principles of chemical exchange saturation transfer (CEST) with hp ^129^Xe as well as considerations regarding different system parameters and saturation-based detection schemes will be discussed as an example for high sensitivity Xe-NMR. Investigation of different members of the cucurbit[*n*]uril family will be elaborated to demonstrate the importance of the exchange kinetics. The translation potential of such host-guest based supramolecular assays to in vivo studies will be duly considered, followed by some suggestions to improve such ^129^Xe-based NMR/MRI investigations in future studies.

## 2. NMR Techniques for Studying Supramolecular Assemblies

### 2.1. General Sensitivity Considerations

**Figure 2:**
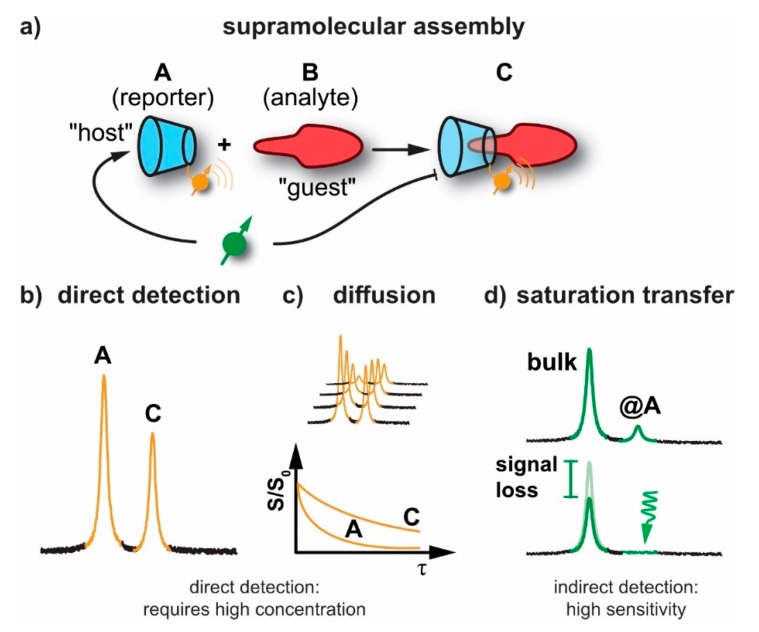
Investigating supramolecular assemblies with NMR. (**a**) Example for a spin label as part of one of the constituents (orange) or a spin label from the free bulk solution pool (green) that can access the binding site of constituent A. (**b**) Direct NMR detection of A yields a second peak upon engaging with B to form C. (**c**) Diffusion-weighted NMR indicates a faster signal decay of the free constituent A than of the complex C with increasing diffusion time τ. (**d**) Saturation transfer NMR rely on detecting spins of free guests (green) in the bulk pool after saturating the magnetization of spins that are transiently bound to the cavity of A. On-resonant saturation causes a signal loss of the bulk pool signal. This process is restricted upon formation of the complex C.

NMR has emerged as one of the most important and versatile techniques in characterizing different materials in chemistry, materials and life sciences. It is applied to polymers, drugs, supramolecular assemblies, and proteins and can report on structure, conformations, and the stereochemistry of organic/inorganic materials. In the most simple case, an NMR-active nucleus of one of the constituents serves as a label and can report the assembly event with another molecule through a change in chemical shift (see Figure 2a,b). Conventional NMR lacks sensitivity due to the rather small spin alignment at room temperature. A quantitative parameter that describes this alignment (for a spin-½ system) is given by the polarization:

$$P=\frac{\left|N_{\mathrm{up}}-N_{\mathrm{down}}\right|}{N_{\mathrm{up}}\;+\;N_{\mathrm{down}}}=\tanh\frac{\hbar \gamma B_{0}}{2k_{B}T}\to \frac{\hbar \gamma B_{0}}{2k_{B}T}$$

where $N_{up/down}$ are the populations of the respective spin energy levels. The above simplification applies to the high temperature approximation (*T*~300 K) and yields very small numbers (O(10^−4^)) due to the fact that the thermal energy linked to the Boltzmann constant $k_{B}$ is significantly larger at room temperature than the Zeeman energy in the numerator that is governed by Planck’s constant ħ. Moreover, widely used ^1^H-NMR of biochemical samples suffers from the background interference arising from abundant nuclei when water has to be used as a solvent. Generally, millimolar concentrations of samples are required to achieve a decent NMR signal for various investigations. This holds in particular for acquiring multidimensional spectra or spatially resolved image (MRI) data. In certain cases, enrichment of different hetero nuclei, e.g., ^15^N, ^13^C, ^31^P, ^19^F, etc. has led to a surge in detection sensitivity of specifically labelled molecules in both solution and solid state NMR. Smaller supramolecular assemblies can be investigated in solution state while solid state NMR (ssNMR) is beneficial for studying larger supramolecular assemblies. The latter applies to biomaterials, proteins, metalloproteins [34,35,36,37,38,39,40]. However, ssNMR is unable to disclose insights linked to dynamics, cavities/pores characteristics and specific interactions that are usually observed in solution phase. 

### 2.2. Diffusion NMR

Diffusion NMR might be considered for characterizing solution-based intermolecular interactions and for studying the dynamics between the molecules of interest and the surrounding media. It is well suited for revealing changes in molecular mass upon supramolecular assembly through reduced, translational diffusion through a magnetic field gradient that induces a signal loss (*S_o_*→*S*, see Figure 2c). It has been applied to polymers [41], zeolites and porous materials [42,43,44], as well as to surfactants [45], liquid crystals, membranes [46] and proteins [47,48,49]. So-called q-space diffusion NMR [50,51] was helpful in obtaining structural information and compartment size of assemblies like biomembranes by sensing diffusion in restricted geometries [52,53]. However, diffusion NMR requires a certain lifetime of the assembly and systems with fast dissociation are not really captured by this method. Similar to above NMR techniques, diffusion NMR also requires usage of high concentration of samples and suffers from relatively low sensitivity and long acquisition times, respectively. 

### 2.3. Detection of Exchange-Connected Pools

To address the aforementioned lack of sensitivity, chemical exchange saturation transfer (CEST) has emerged as a powerful technique such that certain molecules can be studied at relatively low concentrations. Saturation transfer with exchanging nuclei has been originally applied to systems with labile nuclei or for molecules undergoing frequent conformational changes [54]. CEST is effected through the exchange occurring between an abundant (detection) spin pool and a dilute CEST (encoding) pool of an analyte or a specific conformation that is difficult to be detected directly at low concentrations. The latter one carries at least one type of spin label that is saturated selectively while being part of the CEST pool [55]. Supramolecular assemblies can benefit in two ways from this detection principle: a) either one of the constituents has a spin label that undergoes a chemical shift change upon interaction with the other constituent (e.g., through a label that is close to the assembling motif [56]) or b) the confined space of the assembled supramolecular structure is constantly probed by an NMR-active guest such as Xe that changes its chemical shift upon binding and can report on accessibility and changes in exchange kinetics without inducing any change in the supramolecular assemblies themselves (see Figure 2d). 

The CEST-based signal enhancement has been applied to different types of nuclei in order to achieve various applications, e.g., ^19^F- labeled CEST probes and hosts [57,58,59], ^13^C- and ^15^N-NMR of sparsely populated protein conformers [60,61,62,63,64] etc. Additionally, enzyme activity has been detected using catalyCEST agents in which the enzyme acts directly on the CEST agent, e.g., a paramagnetic chelate [65] or diamagnetic species [66,67] (see Section 5.1). A special form of CEST is GEST: in this case, the exchanging spins are part of a guest molecule that has access to a binding site with the purpose of revealing information about the presence and accessibility of the host. It therefore differs from CEST in as much as it involves the exchange of entire molecules and not only of individual nuclei like protons or ^129^Xe. Regarding supramolecular assemblies, the insights provided by CEST with hyperpolarized Xe and ^19^F-GEST NMR will be discussed in more detail in Section 4.

## 3. Cucurbit[*n*]urils and Their Detection with Xe-NMR Spectroscopy 

The use of Xe-NMR for investigating molecular containers such as cucurbit[*n*]urils emerged from the fact that the monoatomic gas undergoes transient binding with various kinds of cavities/binding pockets and thus mimics the behaviour of other guests that engage in non-covalent binding. The noble gas is of particular interest because its large chemical shift range allows identification of resolved peaks from bound spins even for relatively fast exchange rates. 

### 3.1. Synthesis and Properties of Cucurbit[n]urils

The cucurbit[*n*]urils (CB[*n*]s) are a class of macrocyles that exhibit structural resemblance to pumpkins (*Curcurbitaceae*), hence the trivial name of these molecular containers. The building block glycoluril (tetrahydroimidazo-[4,5-*d*]imidazole-2,5-dione) is prepared through a condensation reaction between urea and α-diketones (e.g., glyoxal) under acidic conditions [68,69,70,71,72,73,74,75]. Glycolurils are often utilized as precursors for synthesizing unsubstituted CB[*n*]s. Additionally, both *C*- or *endo*-shaped and *S*- or *exo*-shaped methylene-bridged glycoluril dimers are regarded as the important building blocks for producing different CB[*n*]s and their related derivatives [76,77,78]. CB[*n*]s are synthesized via the condensation reactions between glycoluril and either formaldehyde or paraformaldehyde in acidic (e.g., H_2_SO_4_ or HCl) or refluxing conditions. CB[*n*]s typically comprise *n* = 5–10 glycoluril units that are linked via the two methylene bridges available on each side of the glycoluril. However, the synthesis yields a mixture of CB[*n*]s and CB[6] remains the predominantly isolated product, e.g., as a mix of CB[5] (10%), CB[6] (60%), CB[7] (20%) and CB[8] (10%) [68,79,80]. 

Generally, higher equivalents of formaldehyde to glycoluril units (2:1) are necessary to synthesize various members of the unsubstituted CB[*n*]s (*n* = 5,6,7,8,10,14 etc.) [69]. The proposed steps involved in CB[*n*] synthesis are 1) dimer formation, 2) creation of oligomer ribbons, and 3) the final ring closure [75,81,82,83]. Kim and co-workers tuned the synthesis of CB[*n*]s mixtures by altering the reaction conditions, e.g., glycoluril and formaldehyde condensation in 9 M H_2_SO_4_ at ~75 °C for 24 h, followed by 12 h treatment at 100 °C [79,80]. The as-synthesized CB[*n*]s mixture could be categorized into two parts [84] namely 1) a water-insoluble fractions comprising mainly even “*n*” members such as CB[6], *i*CB[6] (“inverted” CB[6] [70]), CB[8] and CB[5]@CB[10] and 2) a water soluble fraction comprising odd “*n*” CB[*n*] members like CB[5], CB[7] and *i*CB[7], respectively. This solubility effect occurs due to strong and different intermolecular interactions e.g., CH····O hydrogen bonding between the CB[6] resulting in low water solubility. However, in the case of ‘odd’ CB[*n*]s (*n* = 5,7) such interactions are less pronounced (weak interactions) resulting in an enhanced water solubility [85]. Additionally, the inverted members, e.g., *i*CB[6] and *i*CB[7], are also isolated as kinetic intermediates while synthesizing CB[*n*]s in HCl (85 °C) [86]. The percentages of different CB[*n*]s achieved after synthesis is highly dependent upon the solvent and temperature applied during the condensation reaction [79].

The unsubstituted even members of the CB[*n*] family, e.g., CB[6], CB[8] and CB[10], are chemically inert and their poor solubility in common solvents hampers further applications. To some extent, the solubility of these members might be enhanced by utilizing cations or positively charged guest molecules [87,88]. Additionally, solubility issue might be surpassed by synthesizing substituted CB[*n*]s with units that could enhance the solubility of CB[*n*]s in common solvents and also provides a handle that is suitable for further functionalization [87]. To have such interesting properties, a series of substituted CB[*n*]s such as cyclohexano-substituted (CyH)_n_CB[*n*]s (CyH = cyclohexano; n = 1–6, *n* = 5–8 ([89])), Me_12_CB[6] (Me = methyl ([90,91])), (CyP)_n_CB[*n*] (CyP = cyclopentano ([92])), Ph_2_CB[6] (Ph = phenyl ([93])), HMeCB[6] (H = hexa ([94])), TMeCB[6] (T = tetra ([95])), (CyH)_2_CB[6] ([96]) and (Me_2_CyP)_n_CB[6] ([90]), respectively have been synthesized. Cyclohexane derivatized CB[*n*] (*n* = 5–8, (CyH)_n_CB[*n*]s) displayed higher solubility in both water and certain organic solvents compared to other substituted CB[*n*]s. For example, 1,4-DiCyHCB[6] indicated higher solubility in water while preserving similar host-guest properties compared to unsubstituted and almost insoluble CB[6] [97].

CB[*n*]s bind different guests including small organic molecules, amino acids, peptides and proteins [98,99,100,101]. The guest encapsulation is facilitated by ion-dipole interactions and through (non-)classical hydrophobic effects [102,103]. The hydrophobic cavity of CB[*n*]s attracts neutral hydrophobic molecules for encapsulation, while the two identical carbonyl rims entertain positively charged groups, e.g., ammonium groups or other cations. The binding process is also regulated by the size and shape of the guest molecules [102,103,104,105]. An ideal binding of the guest inside CB[*n*]s are anticipated upon reaching a guest volume of ca. 55%. For example, a highest binding affinity of 7.2 × 10^17^ M^−1^ was reported for CB[7] with a diamantane diammonium as guest. Such high guest binding affinity promotes CB[*n*]s applications in drug delivery [106], photodynamic therapy [107,108], diagnostics [28,30,31,109], molecular machines [110], gas sorption [100], artificial ion channels, vesicles [111], supramolecular tandem assays [112], peptide recognition [113], and molecular recognition [114] as well as gas separation/trapping [115,116] etc. 

The encapsulation of different guests by CB[*n*]s is generally investigated using different spectroscopic and analytical techniques. Regarding spectroscopic methods, NMR provides valuable information of the host-guest interaction (inclusion complexes) by means of changes in the observed NMR chemical shifts (see Figure 2a). For example, ^1^H-NMR spectra of CB[5] to CB[8] shows three proton signals pertaining to H_1_, H_2_ present at the equatorial site of the glycoluril unit and H_3_ at the bridges linking two glycoluril units, respectively. A downfield shift of H_3_ (δ_H_ = 5.9 ppm) was observed due to the large deshielding experienced by the H_3_ pointing toward ureido oxygen, while H_1_ and H_2_ appear at 3.3 ppm and 4.6 ppm, respectively, in the gas phase measurement. The observed chemical shifts of the CB[*n*]s protons are independent of the number of glycoluril units present in its isolated state [117]. In the presence of water, the chemical shifts of CB[8]’s H_1_, H_2_, H_3_ were assigned as 3.8, 5.2 and 5.6 ppm, respectively. A large downfield shift was noted for H_1_ and H_2_ in the presence of water but not for H_3_. This clearly indicates the role of the solvent in the determination of the chemical shifts linked to protons available at different positions of CB[*n*]s. The calculated NMR spectra indicated that CB[*n*]-based inclusion complexes upon solvation experience downshifts for the signals from H_1_ and H_2_ while the H_3_ shifts in opposite direction compared to free CB[*n*]s. Additionally, the orientation of the approaching guest to CB[*n*]s also shows a marked impact on the observed chemical shifts of the guest. For example, the ferrocene guest experiences a relatively large upfield shift of its protons upon complexation to CB[7,8] when oriented parallely compared to inclusion complexes displaying perpendicular orientation of the encapsulated guests [118]. 

Although conventional ^1^H-NMR is an essential tool for characterizing host-guest complexation formed by CB[*n*]s, a meager change in the chemical shift (ca. 0.5–1 ppm) often prevents its use in different applications. To overcome such issues, a reporter that can sense the interactions with guests with a well separated NMR chemical shift change (more than 30 ppm) at a significantly lower concentration (µM or lower) of the host will be of more interest. In this regard, hp ^129^Xe-NMR has emerged as a powerful tool for studying such supramolecular inclusion complexes with a pronounced chemical shift difference between bound and free Xe.

### 3.2. Hyperpolarized Xe for Sensitivity-Enhanced NMR

NMR in general suffers from rather low sensitivity compared to other spectroscopic methods. While Xe as a mono-atomic gas with mM solubility in water [119] and other common solvents is a valuable probe to explore binding cavities such as those provided by CB[*n*]s, this limitation of conventional detection becomes especially problematic in combination with the low solubility of certain members of the CB[*n*] family. Long acquisition times become unavoidable even when an overpressure reservoir of pure Xe is available on top of a CB[*n*] solution in a sealed sample. The fraction of ^129^Xe can be enriched to ca. 80% (natural abundance: 26%), but this provides only a ~3-fold improvement in signal amplitude. 

**Figure 3:**
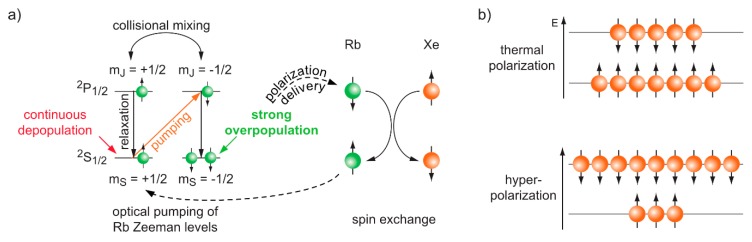
Hyperpolarization of ^129^Xe through SEOP. (**a**) Optical pumping of the Rb electron system and subsequent spin transfer onto Xe in a binary collision. (**b**) Enhanced population difference in the hp spin system compared to the condition with thermal polarization. In this case, the pumped Rb system causes an overpopulation of the upper Xe spin energy level. The eventually detected signal is proportional to the polarization *P* and the spin density.

The relevance of Xe-NMR for interrogating host systems has thus significantly gained since the advent of spin-hyperpolarization techniques that artificially increase the spin population imbalance of the noble gas prior to the NMR measurement. Most studies employ spin exchange optical pumping (SEOP, [120,121], see Figure 3). The hp gas is prepared in a “continuous flow” approach when used for extensive spectroscopy applications. Xe is then bubbled repeatedly as a mix with He and N_2_ into the sample directly after leaving the SEOP setup. One important advantage is that this comes with the option of practically unlimited signal averaging and very reproducible starting conditions in studies where an experimental parameter is incrementally changed over a whole series of acquisitions [122]. 

The key concept of SEOP is to artificially enhance the spin polarization of a precursor system that subsequently transfers its polarization to the detecTable 1^29^Xe nuclei. In brief, this is achieved through (partially) vaporizing a Rb droplet that provides atoms with a single valence electron which can be subject to laser excitation. Using a strong infrared laser beam (795 nm; ~10^2^–10^3^ W cw power) together with optical elements to generate circularly polarized light, the vapour is optically pumped on the Rb D_1_ transition to obtain a strong Rb electron spin polarization. This is achieved by combining the laser illumination of the pumping cell with a static magnetic field in the mT range that is aligned with the laser beam direction. This causes a selective transition from the Rb electron ground to the first excited state with a spin flip according to the selection rules for dipole transitions. The warm temperature conditions (both from heating the Rb droplet and through laser absorption) cause efficient collisional mixing and a subsequent equal population of the spin sublevels of the ^2^*P*_1/2_ state. 

As such, this system would not yet exhibit any spin polarization but quickly undergoes relaxation back to the ground state where only the continuous, selective depopulation of one of the spin states (selectivity is defined through the σ^+^ or σ^−^ polarization of the photons) causes the eventual overpopulation of the other one. It is important that this relaxation step happens radiation free in order to avoid emission of photons with opposite polarization than the ones emitted from the laser system. Such relaxation is achieved through the addition of N_2_ as a quench gas (typically ~10%) that absorbs the energy from Rb and stores it as internal energy of the N≡N system. Moreover, the whole system is usually under overpressure to achieve a better absorption of photons given by the pressure broadening of the Rb D_1_ absorption line. Many setups therefore contain He as the major gas component (~80%) and operate at a few bars over pressure. The SEOP cell is either loaded once with a He/N_2_/Xe mixture or is continuously perfused with this mix.

The polarized Rb vapour serves as a reservoir for transferring spin polarization onto the ^129^Xe nuclear spins for NMR detection. Such polarization transfer occurs in a flip-flop process: The pre-polarized Rb atoms in the vapor state transfer their polarization onto Xe nuclei where both spins undergo a change in the *m_z_* quantum number with a net change Δ*m_z_* = 0. The Rb electron spins immediately undergo re-polarization through photon absorption to serve other Xe atoms. Overall, the polarization transfer depends on the pressure and temperature conditions inside the pumping cell. Modern systems can achieve near-unity spin polarization through careful optimization [123]. An important component of high performance systems is the handling of the energy that is deposited into the N_2_ quench gas through the excited Rb vapor. The laser-produced heat that is generated in the N_2_ reservoir can cause further vaporization of Rb in setups where the alkali metal droplet resides directly inside the pumping cell. This heat typically leads to a process called “rubidium runaway” which is a self-amplifying, detrimental effect that yields poor Xe hyperpolarization because the Rb polarization cannot be maintained at a sufficient level to cause enough net-polarization of the Xe spins. A careful temperature management inside the setup is an efficient way to keep this problem under control [122].

The polarization values that are achieved with modern SEOP setups easily reach *P*~25% in continuous flow [122] and almost *P*~100% in optimized stopped-flow systems. Importantly, this allows one to operate with relatively low concentrations (~µM regime) of CB[*n*]s for direct observation of Xe•CB[*n*] inclusion complexes with ^129^Xe-NMR. Conventional ^1^H-NMR would require significantly higher concentrations to reveal changes in the proton signals.

### 3.3. Delivery and Acquisition Considerations for hp Xe

**Figure 4:**
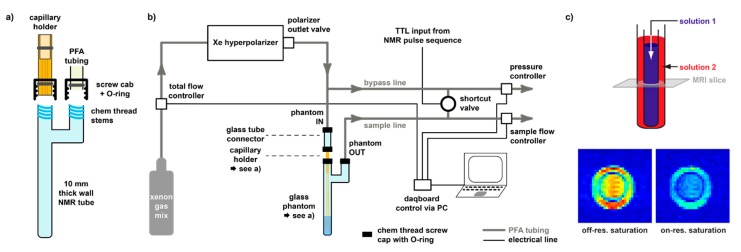
Setup for Xe-NMR of supramolecular systems in solution. (**a**) Sample holder for dispersion of hp ^129^Xe into solution. (**b**) Setup illustrating the interplay between the hyperpolarizer, the connected sample holder, and the valve and pressure control via a PC and the NMR pulse sequence. For more details, see [124]. (**c**) Double phantom setup with exemplary MRI data showing a stronger saturation response for the inner compartment with a Xe host.

It should be noted that the hp state of the spin system is outside the thermal equilibrium and that *T*_1_ relaxation will drive the spin system back to a tiny population difference that is impractical to detect. The favourable conditions thus exist only for a limited time window if no fresh Xe is re-delivered from the SEOP system. The delivery protocol and acquisition techniques have to be adapted to this condition. While continuous-flow operation allows in principle the stepwise acquisition of any complex data set, the repetitive delivery of Xe can become a limiting factor if delicate samples can handle the gas bubbling only to some extent. Typically, the gas is bubbled for 15–20 s at moderate flow rates (ca. 2 mL/min Xe flow rate) into a sample holder (see Figure 4). The NMR pulse sequence is used to trigger the delivery and then allows a time gap for bubbles to collapse before the pulse sequence starts. As Xe is typically still mixed with He and N_2_, the overall gas flow is significantly higher (~100 mL/min) and excessive bubbling should be avoided to keep foam formation in the sample under control. 

The bubbling delivery can become problematic when biological material such as proteins or cells are involved. Strong foam formation can occur as well as accelerated cell death [125]. Synthetic compounds like CB[*n*]s, however, have not shown any incompatibility with extended Xe bubbling. Solution volumes of ~1 mL should be bubbled sufficiently to achieve a Xe-saturated solution. Solvents with higher solubility for Xe (e.g., DMSO) require longer gas delivery. Overall, the repetition time can add up to 30 s or more. Moreover, any magnetization that has been affected by the excitation pulse should be read out as quickly as it further decays. Spectroscopy applications that just screen for pools of bound Xe in CB[*n*]-derived hosts most efficiently use a 90° flip angle, followed by immediate Xe re-delivery.

For some hosts, the repeated delivery of Xe is not beneficial and thus the use of hp nuclei as such is also impractical. The affinity of Xe to CB[5] is an example [126] that is governed by relative slow exchange as given by a release rate *k*_out_ ≈ 10^−1^–10^−2^ h^−1^. This means that on average the host is still occupied for a long time after detection of the Xe guest. Re-delivered Xe would thus have lost its hp state already before entering the host for another detection because the life time of dissolved hp Xe from a He/N_2_/Xe mix is governed by *T*_1_ and amounts to ca. 120 s in water at 9.4 T [127]. 

### 3.4. Direct Detection of Xe•CB[n] Inclusion Complexes

CB[*n*]s with *n* = 5–7 are considered efficient hosts for small guests like ^129^Xe due to their matching cavity size. The commercial availability of them enables a quick evaluation of different CB[*n*] properties, e.g., molecular recognition and inclusion complexes using NMR without any hindrance. However, differential solubility of ‘odd’ (CB[5]: 0.3 mM [128], CB[7]: 20–30 mM [129]) and ‘even’ (CB[6]: 0.02 mM [128,130,131]) in water often requires the addition of solubilizing ions. 

To enable ^129^Xe-NMR applications, a rather loose and reversible Xe binding is a prerequisite for achieving the desired Xe exchange rates, e.g., medium to slow exchange rates. This exchange of Xe is necessary for any measurements with hp ^129^Xe because the enhanced magnetization can be detected only once and needs to be replaced with freshly hp nuclei in cases where signal averaging is required (see Section 3.4). Obviously, such loose interactions are feasible with hosts displaying a cavity volume bigger than Xe itself. However, if the cavity volume is significantly larger than the required space it might lead to very fast Xe exchange and thus no observable NMR signal. This interplay between transient Xe binding, cavity volume, and host isomers/conformations impacts the direct detectability of bound Xe. CB[*n*]s derivatized with water soluble tethers can also display better Xe binding and improved exchange conditions in comparison to poorly soluble, naked CB[*n*]s. This is presumably due to some decelerating effect that the attached units have on the exchanging guest when passing the portal.

**Figure 5:**
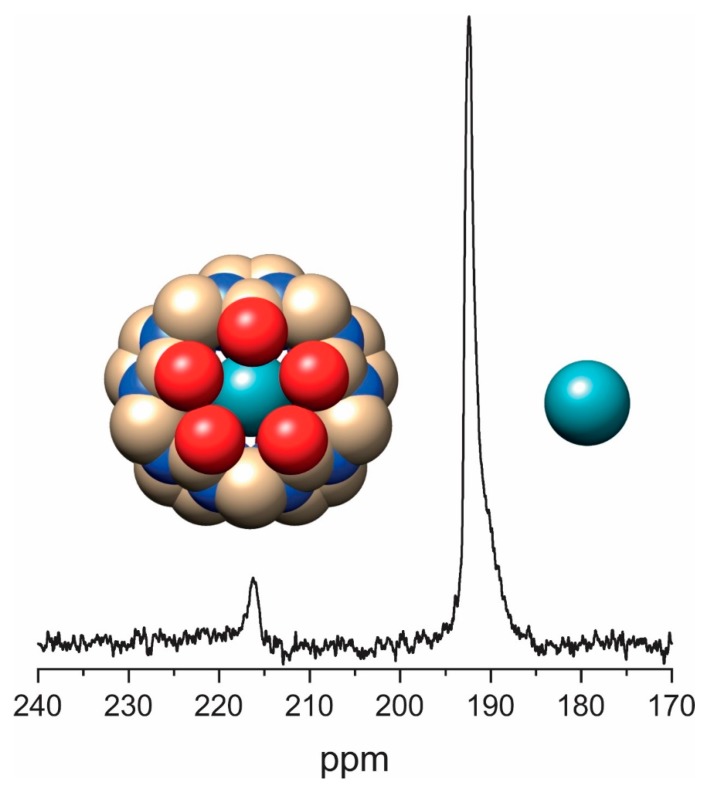
^129^Xe-NMR spectrum of a saturated solution of CB[5] in H_2_O with 13% Magnevist^®^ for relaxation enhancement in solution. The amount of dissolved Xe corresponds to 13.2 mM from 4 atm of Xe on top of the solution. Signal averaging with 5000 FID acquisitions, TR = 45 s, line broadening: 15 Hz (unpublished data from our lab).

With CB[5] being the smallest host of this family, it accommodates ^129^Xe quite tightly with a cavity size of 82 Å^3^. The exchange rate is low enough that no substantial Xe turnover occurs during ^129^Xe-NMR signal acquisition. Together with long *T*_1_ times of ^129^Xe, efficient signal averaging requires the addition of a paramagnetic agent to allow for faster excitation repetitions. Figure 5 shows an example of a saturated CB[5] solution that was acquired with 5000 scans in the presence of 13% Magnevist^®^ in solution and 4 atm of Xe plus 0.5 atm of O_2_ on top of the solution. The sub-millimolar concentration of CB[5] yields only a small signal compared to dissolved Xe (present at ca. 13.2 mM). 

Detectability is better for per-hydroxylated CB[5] ((OH)_10_CB[5]) with a moderate increase in solubility (ca. 0.7 mM at 293 K). Such solubility enhancement promoted the thermodynamics and kinetics-based investigations of Xe binding to CB[5]. Measurement of CB[5] using Xe-NMR revealed spontaneous incorporation of Xe into its cavity with a quite high binding constant and a low in/out exchange rate at 316 K. The ^129^Xe spectrum showed two peaks at 196 ppm (Xe in solution) and ~225 ppm (Xe•CB[5]), respectively. Interestingly, the encapsulated ^129^Xe showed a change in *T*_1_ from 15 ± 3 s to 28±5 s upon thermal cycling between 277 and 315 K. This relatively long *T*_1_ confirms the weak proton-xenon dipolar interaction existing between Xe and CB[5] in which the protons point outwards in addition to contributions from other relaxation mechanisms such as chemical shift modulation [115,132]. The binding constant obtained for Xe bound to CB[5] (*K* = 1250 M^−1^) at 316 K after fitting a model was in good agreement to the value extracted from agas-release experiment. Additionally, the role of water for Xe binding to CB[5] was shown using MeCB[5] at 353 K in water compared to no binding of Xe in the solid phase. It was also found that CB[5] molecules interact with each other differently depending on the cavity being occupied or unoccupied with Xe, thus showing variations in the observed Xe chemical shifts. The Xe binding constant for CB[5] decreases with temperature. 

Using hp ^129^Xe, an NMR spectrum (single scan) of CB[5] (0.25 mM at 316 K) in the presence of less than 1 atm of Xe displays only one signal corresponding to free Xe in solution despite of having a high signal-to-noise ratio. The absence of bound Xe signal is anticipated due to lower exchange compared to Xe relaxation. A long *T*_1_ (660 s) observed for this CB[5] system was similar to that of Xe in pure D_2_O which confirmed no strong superficial binding site existing for Xe with CB[5]. Additionally, subtle differences in gas binding was reported as a function of the methine sites functionalization in CB[5] [133]. To promote high Xe encapsulation by CB[5] in solution, the gas can be trapped at high temperature and might be retained for further use by quickly cooling the solution [126].

The less water soluble CB[6] has received increasing attention over recent years for utilizing it as a host for encapsulating hp ^129^Xe such that its binding capabilities and Xe exchange rates contribute to a growing field of Xe-NMR applications. As its internal cavity (5.8 Å) is a bit larger than that of CB[5], Xe (~4.3 Å diameter) experiences transient binding with medium exchange rates on the Xe NMR time scale. The solubility of CB[6] has to be enhanced such that a direct detection using Xe NMR might be feasible in order to derive the thermodynamic and kinetic parameters linked to its encapsulation process. In this context, Dmochowski and co-workers achieved a higher concentration of CB[6] (>10 mM) by dissolving the commercially available CB[6] in a customized phosphate buffer system with pH 7.2. Using hp ^129^Xe-NMR, it was shown for CB[6] at 5 mM concentration that the bound Xe peak (Xe•CB[6], 121.7 ppm) appeared at 72 ppm upfield shifted from the dissolved Xe peak at 193.5 ppm, respectively. It has to be assumed that the observed Xe chemical shifts are altered due to the presence of monovalent cations at the carbonyl oxygen available at CB[6] portals. 

2D hp ^129^Xe-NMR exchange spectroscopy (EXSY) was utilized to determine the thermodynamic/kinetic parameters linked to Xe encapsulation by CB[6]. The association constant determined for Xe with CB[6] in PBS was found to be in line with values anticipated for Xe-host interactions [115,134]. The rate constants determined using EXSY spectroscopy for association and dissociation were 4.1 × 10^5^ M^−1^ s^−1^ and 840 s^−1^, respectively, which was similar to the values determined for water soluble CB[6] (e.g., 2300 s^−1^ in H_2_O and 310 s^−1^ in 0.4 M Na^+^ solution [115]. To overcome the usage of salt for enhancing CB[6] water solubility, Kim and co-workers synthesized a new water soluble CB[6] derivative (Cy_6_CB[6]) by appending six cyclohexyl (Cy) units at the equatorial positions. This approach tremendously increased the solubility of CB[6] from less than 10^−5^ M to 2 × 10^−1^ M albeit without affecting its cavity dimensions and guest binding properties. The binding constant of Xe•Cy_6_CB[6] was found to be 3.4 ± 0.1 × 10^3^ M^−1^ (determined by ITC) which is comparable to the binding of Xe to water soluble cryptophanes [135]. Using three equivalents of water-soluble CB[6] for encapsulating hp ^129^Xe indicated a bound Xe peak at 97 ppm compared to the free Xe in solution peak at 190 ppm in water. Integration of the NMR signals revealed a binding constant of only ~1300 M^−1^, but still on the same order of magnitude as the one obtained through ITC. Due to line broadening, a more accurate determination of the binding constant of the water soluble CB[6] was not possible. It was thus also measured in the presence of 0.2 M Na_2_SO_4_ and both signals were shifted downfield, presumably due to the interactions between the encapsulated Xe and the cations on the portals [136]. These cations also decelerate the exchange and limit the line broadening, thereby revealing a binding constant for Xe with the water-soluble CB[6] of 180 M^−1^. Thus, the ions reduce the affinity for Xe and yield values achieved for unmodified CB[6] measured either through change in its proton chemical shifts or through another guest competition such as THF, respectively [134,137]. The *T*_1_ relaxation time of Xe in water-soluble CB[6] was calculated to be approximately 40 s and it is much larger than those determined for water soluble cryptophanes [135]. 

CB[7] provides a cavity size of 7.3 Å in diameter and is much more water soluble than CB[6]. Thus, CB[7] was likewise proposed by our group as a potential host for encapsulating hp ^129^Xe in water [138]. It was anticipated that Xe will experience a faster exchange in and out of CB[7] cavity. The additional impact from the accelerated exchange is quite pronounced as demonstrated by the fact that a direct Xe NMR spectrum of CB[7] (250 µM) in H_2_O does not reveal any peak for Xe•CB[7] even after 64 acquisitions with hp ^129^Xe. However, the presence of a second pool that is in fast exchange can be seen by an accelerated decay of the acquired FID signal from the dissolved gas and a corresponding free Xe in solution peak at 190 ppm with an increased line width. A signal for Xe CB[7] was proven only through indirect detection, i.e., HyperCEST-based detection (see Section 4). The observed bound Xe signal is significantly weaker and less intense for both CB[7] and functionalized CB[7] [139] compared to that of CB[6]’s bound Xe peak. This could be partially explained by the larger cavity size of CB[7] compared to CB[6], leading to a weaker Xe interaction and in turn a faster Xe exchange. 

## 4. Saturation Transfer-Based Detection (CEST + GEST)

**Figure 6:**
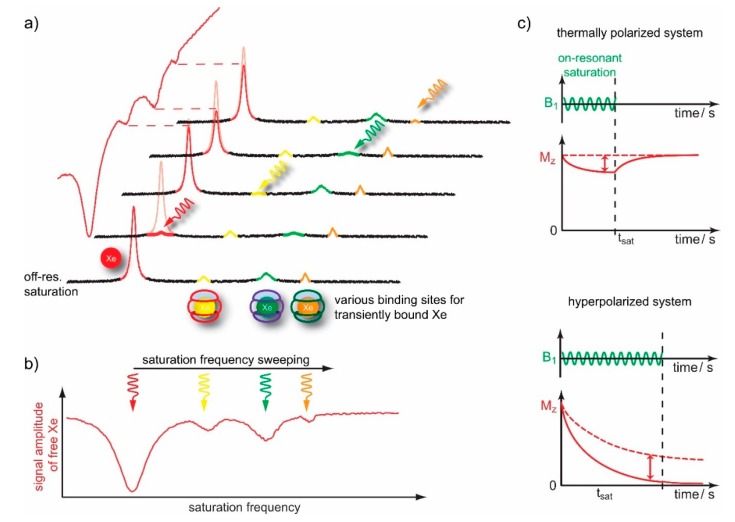
Concept of HyperCEST detection. (**a**) Pseudo-2D spectroscopy data set for preserving the spectral dimension in HyperCEST. (**b**) z-spectrum derived from data in a) (**c**) Time scales in CEST with thermally polarized nuclei (^1^H) and HyperCEST with induced saturation being faster than intrinsic relaxation.

As mentioned in Section 2.3, the efficient exchange of spins between two molecular micro-environments comes with options for CEST sensitivity enhancement. Accessible cavities in either the supramolecular constituents or the complete assembly provide a rather sensitive option to employ exchanging spins and make a more efficient use of the available magnetization. In a typical setup, the pool of unbound spins in solution is usually large compared to the pool assigned to reversibly bound spins. Thus, the exchange can transfer information from the dilute onto the abundant pool that is detected at a much better signal-to-noise ratio (SNR). The combination of hp nuclei with CEST is termed HyperCEST [140] and is especially powerful since it joins two amplification techniques. Hp nuclei can be detected at fairly low spin densities for the bulk pool. HyperCEST therefore allows to keep the host concentration even more dilute. Importantly, the spectral dimension can be preserved in a pseudo-2D experiment for which the saturation pulse is applied at different offsets relative to the frequency of the detected abundant pool (see Figure 6a). The latter one exhibits complete, direct saturation at 0 ppm offset and reveals CEST responses from exchange-connected pools whenever the resonance frequency of transiently bound spins is hit. This data is illustrated in so-called z-spectra where the z-magnetization of the bulk pool is detected after applying RF saturation at various frequencies. As the saturation pulse is usually applied for a time period that is long compared to the residence time of the spins in the CEST pool, many hundreds to thousands of spins can be affected by one host structure. 

Another advantage of HyperCEST is that the driven saturation is not partially counter-acted by *T*_1_ relaxation (see Figure 6b). Whereas systems with thermal polarization (such as Dia- and ParaCEST with exchanging protons and water detection) only achieve a steady state and limited signal loss, hp Xe efficiently “stores” all CEST information in the bulk pool of the dissolved gas. The relatively long *T*_1_ relaxation time of Xe in several solvents allows to apply long saturation pulses and thus accumulate a CEST effect over 20–30 s to detect fairly low concentrations of a binding partner.

Xe is an ideal and versatile nucleus for this technique since it undergoes many transient interactions with molecular partners. Its large chemical shift range enables excellent selectivity when applying the saturation pulse and allows “multiplexing” for working with multiple guests [141]. It is thus much more efficient in detecting relative fast exchanging spin systems because the larger frequency separation between the connected pools represents two important advantages: a) the fast exchange regime in which the pools cannot be resolved any more occurs only for relatively high frequencies (> 10^5^ Hz) and b) it is possible to apply relative strong saturation pulses without causing a “spillover” effect (i.e., the off-resonant RF pulse already directly affects the detected bulk pool). 

### 4.1. HyperCEST Characterics of CB[n]s

The application of HyperCEST to supramolecular constituents like CB[*n*]s was originally motivated by identifying a Xe host with a faster exchange in aqueous solution than the one observed for cryptophane-A (CrA). The latter one is a cage-shaped molecule that had been introduced as a building block for Xe biosensors in MRI applications [140,142,143,144]. While cryptophanes provide a decent binding constant to keep a large fraction of them occupied with Xe in aqueous solution, the exchange rate is suboptimal for achieving a strong CEST effect. It was anticipated that the more open portals of CB[6] actually provide faster exchange and thus this host was chosen for a comparison of its HyperCEST performance with respect to CrA [145]. 

**Figure 7:**
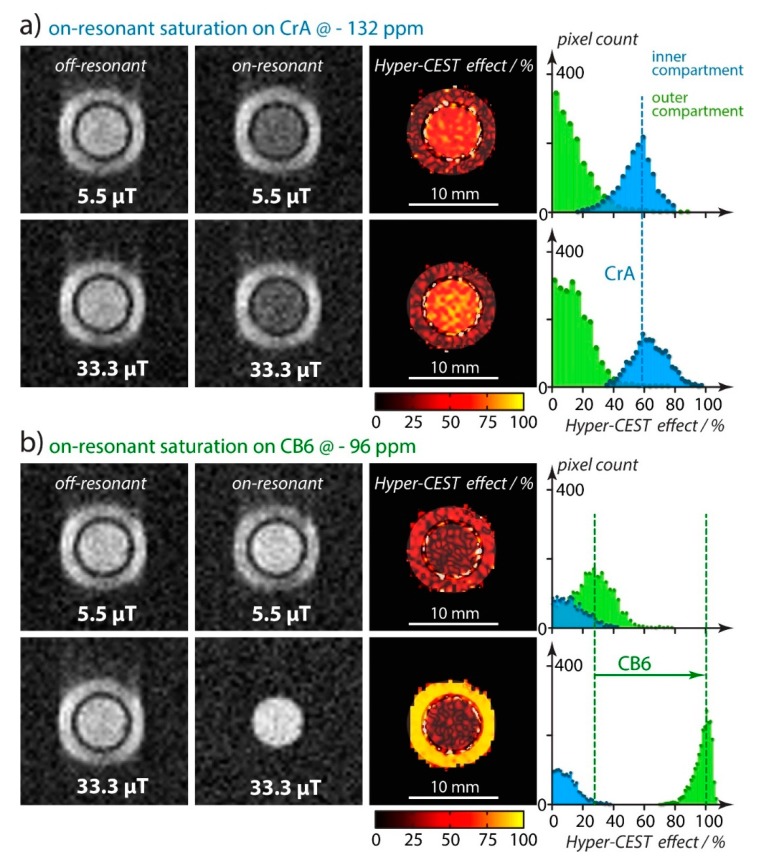
HyperCEST performance of (**a**) CrA and (**b**) CB[6]. This direct comparison of two CEST effects is done with a “double phantom” as depicted in Figure 4c. The histograms represent the distribution of the CEST amplitude throughout the pixels ensemble in the images. The inner compartment with CrA does not improve with stronger saturation power whereas the effect for CB[6] clearly improves for high *B*_1_. Reproduced from [145], published by The Royal Society of Chemistry, licensed under a Creative Commons Attribution 3.0 Unported Licence (20 December 2019).

Indeed, the faster exchange of Xe with CB[6] causes already significant line broadening in conventional direct ^129^Xe detection. At low µM concentrations, the peak of Xe CB[6] is actually hard to identify without substantial signal averaging, even for hp ^129^Xe. Together with the limited solubility of CB[6] in water in the absence of solubilizing cations, the CEST approach is the ideal way to identify the binding of Xe with this host. The z-spectra, however, show a clear response at ca. 100 ppm upfield from the signal of free Xe. The direct comparison with CrA clearly demonstrated the superior HyperCEST performance of CB[6] for imaging applications [145]. However, care has to be taken when comparing results since the benefit of the faster exchange only materializes when suitable saturation conditions are chosen: for cases where the applied saturation power (*B*_1_ of the applied RF pulse) is limited, the slow exchanging system with CrA performs better. CB[6] only surpasses the achieved CEST effect when *B*_1_ is strong enough to achieve sufficient saturation during the much reduced residence time of the Xe spins inside the cavity. This is illustrated in Figure 7 where the applied RF amplitude was increased by a factor of 6 between the CrA and the CB[6] experiment.

This initial HyperCEST MRI study of CB[6] also introduced the gas turnover rate, $\beta k_{BA}$, as a product of the host occupancy β and the release rate *k*_BA_ of Xe from the host (pool B) into the bulk pool (pool A). It is a simple parameter to classify the constant complex association/dissociation of the CEST spin label. The value is derived from quantitative HyperCEST measurements (see Section 4.3) and was determined as 1029%⋅s^−1^ for CB[6] but only 11%⋅s^−1^ for CrA in pure water at room temperature. This is a good example that the sensitivity of HyperCEST clearly benefits from a loose interaction with accelerated exchange (ca. 55-fold faster compared to CrA) despite a ca. 1.7-fold reduced occupancy of the host.

Along this line, the eventually observed HyperCEST signal strongly depends on the exchange conditions provided by the respective CB[*n*] host. Exchange rates of reversibly bound Xe are very susceptible to the cavity size and portal accessibility that the noble gas interacts with [146]. Thus, even the small differences within the subset of CB[5] … CB[7] cover a large range of release rates *k*_BA_ of bound Xe back into the bulk pool. While Xe exchange proved very efficient for CB[6], the rate for CB[5] is too slow to benefit from neither hp nuclei nor an exchange-based signal transfer. Not surprisingly, CB[7] shows the opposite effect and exhibits rather fast exchange. This host was first investigated as an alternative to CB[6] where a higher host concentration was needed due to a large number of competing guests in cell lysate [138]. The low solubility of CB[6] was the limiting factor such that the performance of CB[7] was tested. HyperCEST with Xe CB[7] was studied in the context of a displacement array that is discussed in more detail in Section 5.1

### 4.2. Optimizing CEST Detection

Regarding the duration of the saturation, *t*_sat_, it is important to consider that the CEST effect needs to be “produced” faster than the intrinsic *T*_1_ relaxation causes significant signal loss of the hp nuclei. As illustrated in Figure 6c, the hp magnetization will anyway decay to a non-detectable level if no saturation is applied. Hence, excessive long saturation becomes useless at some point. A mathematical expression for the optimum *t*_sat_ has been derived by Kunth et al. [127] and is a function of *B*_1_, *k*_BA_, *f*_B_ as the fraction of bound Xe, and *T*_1_ in the bulk pool. The contribution from the first three parameters can be summarized in a quantity called on-resonant depolarization rate, λ_on-res_. This is the time constant that governs the RF-induced exponential signal decay when applying the saturation pulse on-resonant. The search for the maximum useful *t*_sat_ anyway only becomes relevant for highly dilute samples where the net saturation transfer builds up very slowly (λ_on-res_→0). In this case, the expression simplifies to *t*_sat_~*T*_1_ of the bulk pool. Hence, the solvent conditions and other factors that impact relaxation of free Xe (protein content etc.) eventually set an upper limit for the useful saturation time. 

As demonstrated by the direct comparison between CrA and CB[6], the faster exchange of Xe with the latter host requires adapting the saturation conditions. Excessive saturation power should be avoided to preserve sufficient spectral resolution (and for respecting RF power application limits for future in vivo studies) but weak saturation is inefficient in affecting the magnetization during the shortened residence time inside the host. Each system therefore has an optimum saturation power up to which the CEST effect for a constant saturation time increases to a notable extent. Further increase of *B*_1_ mainly causes line broadening in the z-spectrum and is not beneficial. Studies have shown that the maximum possible effect is almost achieved for *B*_1_ = 5 *k*_BA_/γ (with γ being the gyromagnetic ratio of ^129^Xe) as this generates 96% of the maximal Hyper-CEST contrast while preserving spectral selectivity. For CB[6] and larger hosts, the exchange rates are easily in the kHz regime and thus this recommended value for *B*_1_ might already be too demanding for cw RF application. The following estimations give an impression about the range of CEST efficiency in cases where reduced powers are necessary:

- *B*_1_ = *k*_BA_/γ yields 50 % of maximum depolarization rate possible, namely λ_on-res_ = (*f*_B_ *k*_BA_)/2;
- *B*_1_ ≤ *k*_BA_/γ yields λ_on-res_ ≅ (*f*_B_ *k*_BA_)(γ *B*_1_)^2^ which is parabolic in *B*_1_ for this low power regime.

When spectral resolution becomes more important due to nearby chemical shifts of exchange-connected pools, one should also consider the width of the signals in the z-spectrum. For a given saturation power *B*_1_, the full width at half-maximum of the CEST response is given by $\Gamma =2\sqrt{\left(\gamma B_{1}\right)2+k_{BA}^{2}}$. This behaviour can be split into two regimes:

- relative strong saturation with *B*_1_ ≥ *k*_BA_/γ shows a linear dependence Γ ≅ 2γ*B*_1_;
- weaker saturation with *B*_1_ ≤ *k*_BA_/γ yields Γ ≅ 2*k*_BA_, which is the minimum possible width that is governed by the Xe exchange rate.

As both λ_on-res_ and Γ increase with *B*_1_, it will be beneficial to evaluate the ratio of fastest depolarization (*i.e.*, a stronger CEST amplitude) and width, λ_on-res/_Γ, as a function of *B*_1_. This ratio illustrates that there is an optimum condition for an intense but reasonably narrow signal. This is reached for a saturation pulse strength corresponding to $\sqrt{2}$ times the Xe exchange rate, i.e., *B*_1_~$\sqrt{2}$*k*_BA_/γ [128].

The application of HyperCEST to certain supramolecular constituents faces challenges regarding rather high exchange rates where even the large chemical shift range of ^129^Xe is not helpful any more to resolve a separate saturation response. Pillar[*n*]arenes are an example of hosts that have been recently demonstrated to form box-shaped structures [147] with matching “lids”. They have also been under investigation for drug complexation and release [148]. As their portals without such lids are rather open, a fast exchange of Xe occurs. However, tuning the exchange rate for such systems with rather loose binding is possible by introducing a co-guest: It was observed that Xe binds to pillar[5]arene in addition to hexane, leading to an upfield shift of more than 75 ppm compared to the external reference solution (Xe solubilized in CDCl_3_) [149]. Moreover, the lipophilicity of pillar[5]arene cavity increases due to hexane encapsulation resulting in a downfield shift for Xe (>10 ppm) compared to Xe@pillar[5]arene. This study motivated the investigation of various derivatives of pillar[5]arene with different counter-ions in water with respect to reversible Xe binding without a co-guest. However, the results pointed towards insufficient deceleration of the exchange. Only a broad response around the direct saturation of free Xe could be observed. Corresponding line broadening in a conventional ^129^Xe-NMR spectrum that decreased with increasing temperature supports the interpretation that this system is already in the fast exchange regime where HyperCEST detection is not feasible. However, Xe-NMR could still give insights into the reversible binding conditions. 

Another important aspect of saturation transfer spectroscopy is the relatively slow point-wise acquisition along the spectral domain. Even the most simple case requires one off-resonant followed by one on-resonant acquisition. This is especially time consuming when working with hp Xe because it requires a fresh re-delivery of the gas when performed the original way. This can be circumvented at least in some cases when the starting magnetization is sufficiently large to share it between the two minimum acquisition by applying a 45° excitation pulse for the off-resonant reference measurement, followed by a 90° excitation pulse for the on-resonant saturation (termed smashCEST, [150]). For obtaining a whole z-spectrum, the acquisition can be accelerated for samples that are isotropic along one spatial dimension. Applying a magnetic field gradient along this dimension in combination with both the saturation pulse and the ADC sampling window for signal readout encodes the spectral information along the spatial dimension [151]. This method is called ultra-fast CEST spectroscopy (UFC). In general, this concept of CEST detection allows significant acceleration of the acquisition and it has been implemented in slightly different forms [151,152,153,154]. Herein, the gradient amplitude determines the spectral width of the z-spectrum to be encoded. Large band widths require strong gradients and are necessary for applications with well separated saturation responses as for ^129^Xe and paramagnetic CEST agents (ParaCEST) in ^1^H detection. The switching of strong gradients may cause eddy currents that yield distortions in the spectra. This can be circumvented by adding a short delay between ramping the gradient and starting the acquisition [155]. For samples that provide long *T*_2_ times of dissolved Xe, it is also beneficial to combine the UFC approach with a spin-echo readout to increase the signal-to-noise ratio [151]. Combining UFC with the smashCEST concept eventually allows the acquisition of a whole z-spectrum with just one Xe delivery. It should be mentioned that the UFC approach can also be applied to imaging data, i.e., obtaining z-spectra from multiple samples next to each other for comparison [153]. Altogether, the in vitro investigation of host systems for reversible binding of Xe and competitive guest can be accelerated significantly to allow for screening of novel supramolecular complexes. Recent examples are the investigation of an entire set of water-soluble pillar[5]arenes [156] and the monitoring of progressive Xe displacement from CB[6] through the enzymatic production of cadaverine [153] (see also Section 5.1).

### 4.3. GEST NMR with ^19^F-bearing Guests 

Exchange-based NMR spectroscopy of CB[*n*]s is not only possible with ^129^Xe. A related approach has been introduced by Bar-Shir and co-workers is based on ^19^F-NMR [146]. Various fluorinated small molecules are known to bind reversibly to members of the cucurbit-[*n*]uril family and can thus be used for saturation transfer spectroscopy.

**Figure 8:**
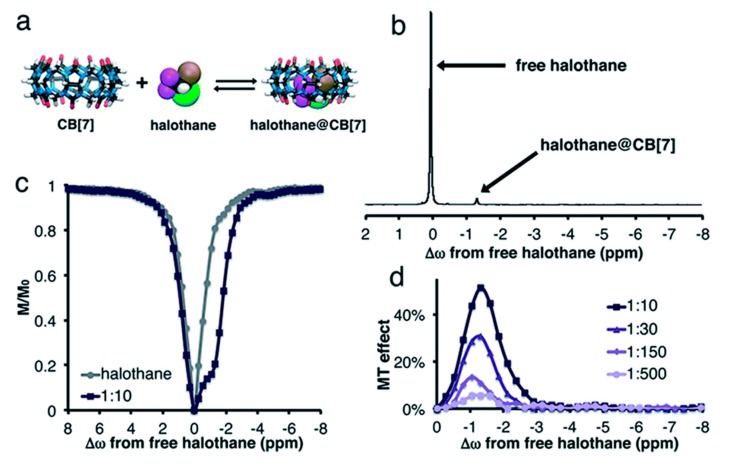
Example of ^19^F-GEST spectroscopy with CB[7]. (**a**) Water-soluble halothane forms an inclusion complex with CB[7]. (**b**) The exchange of halothane is much slower compared to Xe•CB[7], as seen by the appearance of a second peak in the direct spectrum. (**c**) Addition of CB[7] to dissolved halothane causes a pronounced saturation transfer response that overlaps with direct saturation of free halothane. (**d**) Even a 50-fold dilution of the CB[7] fraction keeps the supramolecular complex detectable. Reproduced from [58], published by The Royal Society of Chemistry, licensed under a Creative Commons Attribution 3.0 Unported Licence 20 December 2019).

One example is halothane (2-bromo 2-chloro-1,1,1,-trifluoroethane) with three equivalent ^19^F nuclei that sense the interaction with CB[7] and CB[8] in a GEST experiment (see Figure 8). The larger size of halothane compared to a Xe atom shifts the sensitivity towards larger guests [58]: while Xe is already in too fast exchange to yield a signal in direct detection when bound to CB[7], halothane still yields a resolved peak for CB[7] but not for CB[8]. The presence of ions does impact the exchange and measurable GEST effect as in the case for Xe HyperCEST that senses deceleration by ions interacting with the portals. In both cases, the chemical shifts can also change quite significantly when buffer solutions are used instead of pure water. As much as ^129^Xe HyperCEST is helpful to detect the poorly soluble CB[6], the same holds for halothane and CB[8] which is also only soluble at low µM concentrations. Other supramolecular containers beyond CB[*n*]s that have been investigated with GEST NMR are octa-acid [59] and bambus[*n*]urils [157]. The latter ones were studied with BF_4_^−^ as a guest.

Both ^129^Xe HyperCEST and ^19^F GEST NMR allow a quantitative analysis of z-spectra to obtain absolute values for various parameters that characterize the 2-pool system. The analysis relies on solving the Bloch-McConnel equations for exchanging spin pools under the influence of a saturation pulse. Details will be discussed in the following section. 

### 4.4. Quantitative Saturation Transfer Analysis (qHyperCEST)

Although the HyperCEST detection sensitivity can reach picomolar concentration ranges, a z-spectrum per se does not provide direct quantitative information about the inclusion complexes. An important aspect of z-spectra is that the intensity of the CEST response does not scale linearly with the host concentration. At high concentrations and/or sufficiently strong saturation pulses, the system quickly reaches a state where the host is re-loaded with already saturated magnetization. This is called “back exchange” and becomes increasingly important when exceeding CEST effects of more than 30%. The theoretical shape of a HyperCEST spectrum was predicted by Zaiss et al. [158]. A set of HyperCEST acquisitions at varying saturation pulse power and duration with global fitting for quantitative analysis is called qHyperCEST [159]. 

In qHyperCEST measurements, multiple z-spectra are acquired at different saturation conditions and fitted with the full Hyper-CEST (FHC) solution [158]. The entire set of obtained quantitative parameters comprises the ratio of bound to free Xe (*f*_B_), the Xe release rate (*k*_BA_), the chemical shifts of free and bound Xe (δ_A,B_), the Xe association or binding constant (*K*_A_) and the Xe host occupancy (*β*), respectively. The first test case was the cage-like molecule CrA-ma (11 µM) in H_2_O with *K*_A_ = 850 ± 250 M^−1^; *β* = 29 % and *k*_BA_ = 38 ± 6 s^−1^, implying that CryA-ma is in the slow exchange regime on the NMR time scale [159].

The stability of qHyperCEST analysis was also tested by simulating conditions including noise. For 10% noise, the analytical FHC model performed well compared to the conventional numerical solution, i.e., the (Bloc-McConnell) BM model. However, it is important to apply saturation powers that cover a certain dynamic range of CEST effects: the analysis returns ambiguous results for cases where changing *B*_1_ does not yield clear changes in CEST amplitude but only in the width of the peak. 

One of the aims of using qHyperCEST is to determine the Xe exchange and binding parameters for conditions that are below the detection limit of directly detected ^129^Xe-NMR. This holds especially for the CB[*n*]s with their relatively fast Xe exchange in the absence of cations. qHyperCEST was applied to CB[6] in H_2_O where a direct ^129^Xe-NMR spectrum of CB[6] at 3.4 µM without salt addition yields no signal for bound Xe. Contrarily, using HyperCEST at varying saturation conditions revealed a CEST peak (−95.6 pppm) indicating Xe CB[6] inclusion complexation. qHyperCEST analysis returned the following values: *k*_BA_ = 2100 ± 300 s^−1^, *K*_A_ = 2500 ± 400 M^−1^, and *β* = 49%. This was in agreement with the values reported for water-soluble CB[6] derivatives. Comparing the Xe-CB[6] system to Xe-CryA-ma indicated that CB[6] is in an intermediate exchange regime on the NMR time scale. As mentioned in Section 4.1, it can be useful to define the gas turnover rate to compare and classify different hosts (e.g., CryA-ma and CB[6]) on the HyperCEST performance scale. This can be calculated directly as *β*⋅*k*_BA_ from qHyperCEST analysis and is the maximum depolarization rate per host molecule for a given Xe concentration. 

It should be mentioned that exchange-connected pools of Xe can also be investigated beyond a saturation transfer experiment. The common aspect of such detection schemes is manipulation of one pool, followed by observation of the system during equilibration of this perturbation. This has been demonstrated with different implementations for CrA [160] but also as an inversion-recovery experiment for CB6 [161] albeit with relatively high concentration of the host such that the spin pools were of comparable size.

## 5. Cucurbit[*n*]uril-Based Supramolecular Systems

### 5.1. Displacement Assays

The concept of displacement assays has been widely used for supramolecular systems with competing guests. As an example, supramolecular tandem assays (STAs) based on fluorescence detection have gained much attention due to their label-free approach involving less or no further preparation/separation steps. As described in the introduction, STAs for optical detection involve reporter pairs comprising a macrocyclic host and a fluorescent dye where the binding of the latter one to the host is being suppressed by the stronger binding partner. The reaction course is thus monitored through the change in fluorescence upon displacing the dye. Such STAs can serve as a simple, inexpensive, sensitive, and label-free method for continuous monitoring of different enzymatic reactions [27]. Although the STA approach is quite powerful, it requires sufficiently translucent samples [28]. Conversely, NMR is the method of choice for investigating opaque samples with unlimited penetration depth. 

Extending the displacement assay concept from fluorescence to NMR is therefore an appealing concept to ensure that the substrate remains unmodified and only the product induces a signal response upon its interaction with the host. This concept became achievable through ^129^Xe HyperCEST NMR [138]. A supramolecular enzymatic assay is effectively monitored after the enzyme converts the substrate into a product where the latter’s high affinity displaces Xe from the host. This leads to reduction in the observed ^129^Xe HyperCEST response and was first implemented for the decarboxylation of lysine through LDC that yields cadaverine (Cad) [138]. 

One advantage is that a loss in enzyme activity is prevented by preserving the native state of the utilized substrate. While the displacement assay works straight forward under idealistic in vitro conditions in buffer, a promiscuous behaviour of CB[6] imposes challenges under more realistic conditions that include ions or other guests that interact with the portals or the cavity, respectively. Competing guests in, e.g., cell lysate are already outcompeting Xe at the maximum achievable concentrations of CB[6]. The assay was thus implemented with CB[7] that can be dissolved at higher concentrations. However, the accelerated exchange of Xe yields a rather inefficient CEST effect under these conditions and the z-spectrum is characterized by a broad direct saturation response for unbound Xe. In fact, the origin of the weak CEST response with regard to the broad direct saturation response has been revisited recently [139] (see Section 5.5). A stronger signal was in fact obtained through slightly off-resonant magnetization transfer instead of a unique CEST response. This effect can be used both for spectroscopic information and diagnostic MR imaging for delineating the enzyme-active from the enzyme-negative sample [138].

**Figure 9:**
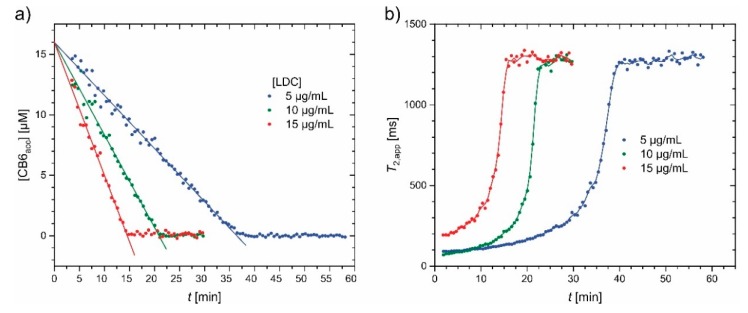
Monitoring LDC enzyme activity with HyperCEST. (**a**) The amount of Xe-accessible CB[6] decreases as the enzyme converts lysine into cadaverine. The CEST effect, which is finally lost, allows to directly derive [CB6_acc_] when the starting concentration is known. (**b**) Likewise, the decreasing amount of Xe that can engage in fast reversible binding causes a loss of exchange-induced *T*_2_ effects. *T*_2_ increases until only free Xe in solution exists. The kink in the curve yields the time when 16 µM of CB[6] are completely blocked by cadaverine. Reproduced with permission from [162], Copyright © 2017 John Wiley & Sons, Ltd (15 December 2019).

A follow-up study with CB[6] (and no lysate) was performed in a time-resolved approach where the chemical shift information is encoded with the UFC method (see Section 4.2) in the presence of a magnetic field gradient. Temporal resolution of ca. 30 s allowed to follow the onset of lysine decarboxylation. The knowledge about the line shape in the z-spectrum was used to rescale the CEST responses according to exponential Lorentzians [158] and quantify the amount of Xe-accessible CB[6] ([CB6_acc_]) as a function of time. Based on the fast dissociation of Xe from the host and efficient association of any newly produced Cad with CB[6], several assumptions can be made that simplify the qHyperCEST evaluation. The experimental results for probing a 6 mM reservoir of Lys with 16 µM of CB6 confirmed the linear progression of the reaction as only the conversion of the initial 2.6‰ of the substrate has been monitored. The overall reaction velocity depends on the enzyme concentration (see Figure 9a) and manifests as differently fast changes in the z-spectra.

It should be noted that changes in the accessibility of Xe to a binding site with a large chemical shift difference can also cause significant changes in the apparent *T*_2_ relaxation time of ^129^Xe. Formation of the Cad CB[6] inclusion complex prevents Xe from experiencing frequent changes in resonance frequency. As long as many CB[6] cavities are accessible, the large chemical shift range of Xe induces a rapid loss of phase coherence for nuclei that frequently swap between two different environments. This represents an alternative detection method of displacement assays. The concept of so-called *T*_2ex_ agents is also known in ^1^H-NMR from paramagnetic chelates that transiently coordinate H_2_O [163]. For Xe, however, the same effect can be observed with a diamagnetic system where apparent *T*_2_ times are drastically shortened as long as the atoms have access to a binding site (see Figure 9b). Once the accessibility changes like in the case of CB[6] being increasingly blocked through binding of Cad, transverse relaxation of Xe as it manifests in CPMG echo trains increases. Overall, 16 µM of CB[6] could reduce the transverse relaxation time of dissolved ^129^Xe ca. 13-fold down to 100 ms in aqueous solution [162].

Enzyme activity detection with supramolecular assays and Xe has several advantages compared to ^1^H-NMR approaches. While the latter one also offers CEST detection options (termed catalyCEST [55]), the lower efficiency of CEST with thermally polarized nuclei requires that a substantial fraction of the substrate needs to be converted into the product. CatalyCEST has been implemented with temporal resolutions ranging between 3.6 [164] and 7.5 min [67] while requiring relatively high substrate concentrations (ca. 50–60 mM). This clearly illustrates the advantages of the ^129^Xe HyperCEST approach with its higher sensitivity and better temporal resolution. Additionally, considering only the start of the reaction (a µM fraction of the mM substrate pool is converted) yields a linear signal behavior over practically the entire dynamic range with the corresponding host concentration. Moreover, catalyCEST still requires the substrate to be provided with a CEST label. This chemical modification can interfere with the enzyme activity. Recent enzymatic CEST platforms rely on salicylic acid and include two CEST sites in the substrate with one of them being lost in the product [67]. Such systems experience different limitations such as reduced activity upon coupling to the reporter moiety (salicylic acid) and less stability in enzyme-free solution [164]. Different CEST agents have been introduced for detection of various types of enzymes, e.g., esterase [165], sulfatase [66,166], transglutaminase [65] and glucuronidase [164] etc. However, many enzymes display reduced activity once its original substrate is coupled to the CEST unit. Therefore, an approach in which the enzyme acts on the native substrate without hindering the former’s activity will be highly desired in enzyme based studies.

Another system that relies on the observation of Xe displacement for detecting the presence of an analyte is based on CB[7] which has an affinity for phenylalanine (Phe). The interaction between this amino acid and the molecular container was first described for insulin [113] and the amyloidogenic peptide Aβ40 [167] using methods other than Xe NMR. The affinity is sufficient to disturb the binding of Xe and thus detect the presence of Aβ40 through Xe MRI [168]. This study could demonstrate that Xe binding to an alternative host such as CrA is not affected by the presence of Aβ40. Hence, an approach with a dual host readout provides an internal control for the loss of the CB[7] CEST response. Due to the different exchange behavior of Xe in either CrA or CB[7], balanced saturation transfer conditions are required to retain spectral sensitivity when detecting both hosts in the same setup. 

### 5.2. Molecular Relays with Two-Faced Guests

A special implementation of the displacement concept can be achieved with molecular guests that have two different binding motifs for engaging with different hosts. As such, this method to control and monitor the sequential interaction of different molecules in solution is of general interest as it assists the generation of molecules with useful properties. Applications include, e.g., enzyme- and small-molecule mediated tandem reactions, as well as drug delivery and nanoscale architectures [169,170,171]. Using structurally rigid CB[6], Dmochowski and coworkers introduced a “programmed molecular relay” concept involving three sequential recognition events to specifically detect a protein in solution using hp ^129^Xe-NMR. This concept mainly relies on a two-faced guest (TFG) that binds with intermediate affinity to CB[6], but also with its opposite face at higher affinity to a protein target of interest. To build such TFG, butylamine was chosen as it displays intermediate affinity for CB[6] (*K*_A_ = 2.85 × 10^5^ M^−1^ in PBS at 300 K) in addition to carbonic anhydrase II (CAII EC4.2.1.1) binding moiety *p*-benzenesulfonamide. Both building blocks were tethered together in order to generate four TFGs with varying length and chemical structure of the linker. CAII was chosen in this study since it is a model enzyme of biomedical relevance [172] with a single binding site and several inhibitors for CAII are also known [173]. The active site of CAII resides at the base with a size of approximately 15 Å as deep conical pocket that differentiates between different TFGs albeit the varied inhibitors (TFGs) share the same Zn^2+^ targeting moiety [174]. The ITC-based binding studies performed between CB[6] and four TFGs indicated binding constants in the range of 1–3 × 10^5^ M^−1^ at 300 K in PBS buffer containing 1% DMSO. Conversely, binding between TFGs and CAII in the same buffer conditions showed the highest binding for the TFG comprising an acid derivative of a butylamine tail linked to *p*-amino benzenesulfonamide via a -CH_2_COOH functional group. The crystal structure of CAII with this TFG (lacking an ethyl linker) revealed that the butyl tail is less solvent-accessible in the complex and supports the interpretation of a presumably lack of any steric clashes upon formation of a stable ternary structure between CAII, TFG and CB[6]. 

For the intermediate step in the programmed molecular relay, the CB[6]-TFG complex was incubated with CAII for 20 min while monitoring the hydrolysis rate of the substrate (*p*-nitrophenyl acetate (*p*NPA)) by absorption measurements in solution. The TFG with higher affinity also exhibited a stronger CAII inhibition. Once complexed to CB[6], the TFG inhibited the CAII only slightly less than TFG alone, thus confirming that the TFG shuttles between CB[6] and CAII. This shuttling is thermodynamically controlled as evidenced through titration of CB[6] with CAII, respectively. This molecular relay concept was extended to NMR in order to generate a “turn-on” strategy-based Xe NMR biosensor. ^129^Xe-NMR of CB[6] (1 µM) in PBS revealed two peaks at 193 ppm (^129^Xe_aq_) and 122 ppm (Xe•CB[6]), respectively. Addition of TFG (4 µM) reduced the bound Xe CEST response at 122 ppm owing to the intermediate TFG binding to CB[6]. Introducing CAII (4 µM) into the solution almost restored the peak at 122 ppm, thus demonstrating that the TFG was sequestered by CAII addition. The molecular relay was also implemented using commercially available pentylamine biotin (*p*AB) as TFG that binds CB[6] less tightly (*K*_A_ = 4240 M^−1^) than its target protein avidin. Addition of 50 µM commercial TFG to CB[6] (1 µM) fully ‘turned off’ the Xe@CB[6] peak, while it was completely ‘turned on’ upon addition of avidin (50 µM), thus conforming the reprogramming of the CB[6]-TFG relay for assaying a wide range of biomolecules in solution. Extending the relay to cellular environments (e.g., *E.Coli* over-expressing recombinant CAII) surprisingly indicated that CB[6] (16 µM) gave a Xe•CB[6] signal in spite of CB[6] experiencing non-specific binding from different components of bacterial cell lysates (as reported in [138]). Subsequent introduction of the TFG (4 µM) to a control sample (non-transformed *E.Coli*) greatly reduced the CEST response from 16 µM CB[6] compared to lysate from CAII-overexpressing *E. Coli* in which the original CEST signal intensity was preserved. These findings show the ability of CB[6]-based detection in identifying a specific protein within a complex mixture [175]. 

Similarly, in another version of a molecular relay, our lab utilized a putrescine derivative of aminomethyladamantane (AMADA-Putr) [176] as TFG that can interact differently with the two Xe-binding host systems CB[6] and CB[7] via ternary complex formation. This generates a “turn-on” strategy based Xe biosensor. The binding of this TFG to CB[6] is effected through the putrescine unit while a strong interaction with CB[7] is enabled by the adamantyl unit. ^129^Xe HyperCEST was utilized to monitor the kinetics of the molecular relay-mediated transition occurring for AMADA-Putr between its complexed state in CB[6] and CB[7]. The CB[6] (4 µM) accessibility for Xe was blocked initially by providing AMADA-Putr (4 µM) leading to a complete loss of the CEST response for Xe@CB[6]. Subsequent addition of CB[7] (5 µM) to the above inclusion complex in solution resulted in an immediate CEST response pertaining to Xe@CB[6]. The course of the reaction indicates that CB[6] was released over time from the ternary complex such that the former is available for inclusion complexation with Xe. Even though CB[6] binds Xe after its release from ternary complex, its CEST signal is not completely recovered due to a remaining small fraction of the ternary complex [177].

### 5.3. Molecular Rotaxanes

Molecular rotaxanes are a special case of supramolecular assemblies where the constituents are mechanically interlocked. Changes in the interlocked state can be triggered by an external stimulus. ^129^Xe-NMR has emerged as a tool to investigate such rotaxanes since the Xe can act as a guest that probes the interactions occurring within the rotaxane. The word “rotaxane” was derived from Latin meaning ‘wheel and axle’ and it is used to describe the compound comprising a linear guest or rod-like unit and a cyclic host or bead-like unit. Both species are interlinked non-covalently in a threaded fashion. The units in a rotaxane are held together by sterically bulky groups (so-called stoppers) to ensure the axle does not pass through the ring component [178]. Rotaxanes without stoppers at both ends are termed ‘pseudorotoxanes’ while rotaxanes appended with one stopper are named ‘semirotaxanes’, respectively [179].

Regarding rotaxane generation, CB[6] was utilized extensively as the preferred host for accommodating the positively charged ‘thread’ component. Additionally, CB[6] entertains hp ^129^Xe after the onset of a specific event that triggers cleavage of the ‘thread’ from the rotaxane. As a side effect, CB[6] solubility in solution can be somewhat enhanced. The molecular ‘threads’ involved in rotaxane formation are basically designed by considering the molecular recognition properties of CB[6], e.g., an affinity for positively charged guests. Such strategy was employed to generate a chemically activated CB[6]-rotaxane platform for ‘turn on-off’ ^129^Xe biosensors [180]. In this strategy, the stopper (‘dumbbell’) on the rotaxane blocked ^129^Xe accessibility to CB[6] in a ‘turn-off’ state. Inducing cleavage at the respective site in the ‘thread’ resulted in a ‘turn-on’ state of the rotaxane in which CB[6] was freed to facilitate ^129^Xe binding. The stoppers such as pyrene-functionalized 2-azidoethylamine (PyAA^+^) and an adamantyl-ester functionalized propargylamine (*AdPA*^+^) were linked together by CB[6]-catalyzed azide-alkyne 1,3-dipolar cycloaddition. Usage of β-cyclodextrin as caps was necessary for solubilizing the two stoppers in aqueous solution [181,182,183]. ^129^Xe NMR of the fully assembled rotaxane (100 µM) showed no dedicated Xe@CB[6] signal, thus suggesting that CB[6] remains in the ‘turn-off’ state. The rotaxane was ‘turned-on’ through base-catalysed ester hydrolysis (e.g., with LiOH for 8 h) after which a semi-rotaxane was formed as a product. This semi-rotaxane displayed suitable Xe binding and exchange kinetics for producing a HyperCEST response.

Similarly, above rotaxane-based approach was extended for selective and sensitive detection of a protease activity (MMP-2) using ^129^Xe HyperCEST NMR. In this approach, one stopper (PyAA^+^) was conjugated to (5,6)-carboxytetramethylrhodamine (TAMRA) as the complementary stopper via an axle comprising a PLG-LAG peptide sequence. The latter is recognizable by matrix metalloprotease-2 (MMP-2). CB[6]-catalyzed azide-alkyne 1,3-dipolar cycloaddition was helpful for installing the CB[6] onto the axle. The ‘turn-off’ state was proven by observing no significant Xe NMR signal for the full rotaxane, thus suggesting that the stoppers remain in place and keep every unit intact. The ‘turn-on’ state of the rotaxane at 5 µM concentration was attained after cleaving it for 24 h by MMP-2 (5 nM). The cleavage led to CB[6] release from the rotaxane axle as revealed by the appearance of a CEST response from Xe•CB[6] (15% CEST effect) [184]. This elegant rotaxane-mediated CB[6] HyperCEST response manipulation can be used for applications in drug delivery and targeted multimodal imaging.

**Figure 10:**
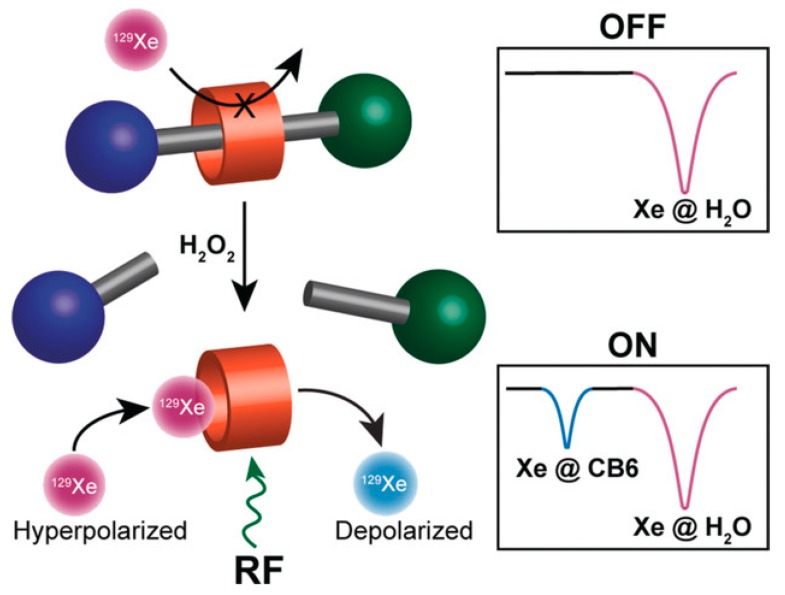
Principle of an activatable rotaxane system for HyperCEST detection. The inact rotaxane allows no access for Xe into the CB[6] unit and thus gives signal only for direct saturation of unbound Xe. The H_2_O_2_-cleavable unit enables a break of the axle and subsequent release of CB[6] to observe a CEST response from reversibly bound Xe. Reproduced with permission from [191], Copyright © 2019 John Wiley & Sons, Ltd (20 December 2019).

Recently, a CB[6]-based rotaxane approach was also used for studying extracellular H_2_O_2_ generation at low physiological levels (0.5–50 µM) which is manifested in different diseased states [185,186]. The rotaxane as illustrated in Figure 10 was composed of a *p*-xylenediamine moiety (a weak guest for CB[6] with *K*_A_ = 5.5 × 10^2^ M^−1^) and an aryl boronic acid group (a H_2_O_2_ cleavable cap), respectively. A non-responsive control rotaxane was generated by tethering it either to a fluorophore or maleimide to facilitate fluorescence measurements and/or for further functionalization to biomolecules of interest. The ‘turn-on’ state of the rotaxane (25 µM) was achieved after cleavage (1 h) initiated through addition of H_2_O_2_ (50 µM), thereby leading to an apparent maximum saturation transfer of about 25% from Xe•CB[6]. Mass spectrometry indicated that H_2_O_2_ does not influence the structural integrity of CB[6] and the cleaved axle. As a proof of concept for rotaxane functionalization, maleimide was linked to cysteine residues available on the vascular cell adhesion molecule (VCAM)-1 binding peptide or to a tobacco mosaic virus (TMV) protein-based nanoparticle. This protein assembly could be advantageous for drug delivery and imaging applications [187,188]. VCAM-1 peptide was completely modified while the TMV was purposely modified at 30% to have 10 rotaxanes installed per TMV disk. To check the feasibility of H_2_O_2_ detection in cellular environments, HEK293T cells were exposed to tumor necrosis factor alpha (TNF) to enhance the cellular H_2_O_2_ production [189,190]. To do so, HEK293T cells were treated with 40 µg of TNF (TNF+) or remained untreated (TNF-) and incubated for 6 h at 37 °C. The TMV-rotaxane conjugate (final concentration of 1 µM) was suspended in the supernatant solution and the H_2_O_2_ mediated cleavage reaction was monitored by ^129^Xe HyperCEST over several hours. Untreated cells (TNF−) remained in the ‘turn-off’ state even after 8 h, while a stable ‘turn-on’ response was observed for TNF+ cells after 2 h and lasted until 24 h. This rotaxane-based approach could monitor the low micromolar H_2_O_2_ levels in cells using ^129^Xe HyperCEST NMR [191,192]. 

### 5.4. Supramolecular Assemblies Between two Hosts

The previous sections described typical host-guest interactions with a clearly assigned role of each constituent. However, it can also occur that two host-type structures interact with each other. The spontaneous self-assembly between different hosts possessing different properties has been tested in aqueous conditions in order to solubilize aless soluble host through a more water soluble counterpart, e.g., cyclodextrins (CD) for solubilizing other hosts like CB[6], dibenzo-18-crown-6 or *p*-*tert*-butylcalix[4]arene [193].The functional groups decorating the host surface or rim play a pivotal role in such host-host complexation. Majorly, such associations are driven through the electrostatic, ionic-dipole, charge-charge, π-π, or van der Waals-based interactions between these hosts. Such complexation between unsubstituted hosts leads to weak interactions and complexes with low stability constants. Additionally, self-assembly between two hosts having different intrinsic properties might lead to generation of host systems or supramolecular structures/architecture with interesting hybrid properties. To enable strong interaction between the two hosts of interest, usage of a molecular thread is inevitable as it is necessary to interlink both hosts in the as-formed inclusion complexes. As such, this can resemble a rotaxane architecture, but with the feature of accommodating another guest. The choice of suitable molecular threads that might be entertained as guests by both hosts is highly dependent upon the intrinsic molecular recognition pattern of each individual host involved in the complex formation. Sometimes, the inclusion complexes containing a molecular thread are generated step wise rather than a spontaneous self-assembly of each component together.For instance, CB[6] strongly interacts with dihexylammonium (DHA) followed by complexation of the latter one to CD to form a ternary complex of CB[6]-DHA-CD (1:1:1) [194]. Another approach would be to interconnect two hosts via a short linker such that a fluorescent host species might be utilized for detecting organic analytes in solution [195]. Such interconnected hosts are useful in orienting the incoming fluorophore for intramolecular inclusion and also enlarge the hydrophobic surface of a host for binding analytes e.g., β-cyclodextrin-calix[4]arene couples linked via a phenyl ring [196].

Likewise, a spontaneous self-assembly of two unsubstituted hosts in water without any elaborative synthesis or derivatization of hosts would also be of interest. Recently, it was shown that CB[7] instantaneously self-assembles upon the addition of water soluble 4-sulfocalix4]arene (SC[4]A) leading to the formation of microcrystals in near-quantitative yield (>90%) in water [197]. This suggests that it was a strong outer-surface interaction of CB[7], i.e., π-π interaction of carbonyl bonds of CB[7] with aromatic rings of SC[4]A in addition to ion-dipole interaction between the electropositive outer surface of CB[7] with the electronegative sulfonyl groups of SC[4]A, respectively [198,199,200]. The as-formed inclusion complex (3:1) between CB[7] and SC[4]A was found to accommodate some volatile compounds e.g., polychloromethanes and also selectively absorbs certain organic dyes e.g., pyrenemethanamine hydrochloride, umbelliferone etc. to yield light emitting solid fluorescent materials [197]. However, an investigation with ^129^Xe NMR remains to be done but could give meaningful insights into the cargo release. 

Along this line, our lab has investigated the option of applying Captisol^®^ (sulfobutylether derivatized β-CD (SBE-β-CD or CS)) to solubilize the less soluble CB[6] in water (up to 200 µM; unpublished data). This enhanced solubility was suggested to happen through the external interactions occurring between CB[6] and CS promoted through ‘supramolecular-based positive cooperativity’. Such systems are generally studied using ^1^H and diffusion NMR at higher concentrations (mM range). Conversely, we have studied such systems with ^129^Xe HyperCEST by relying on the intrinsic molecular motifs of a host for complexing its counterpart. CS with different degrees of substitution (DS) was used, e.g., CS DS6 and CS DS4 within the concentration range of 20–1000 µM for evaluating its impact on the Xe•CB[6] signal arising from the ternary complex formation (Xe•CB[6]-CS). Measuring CB[6] (5 µM) in the presence of CS DS6 (100 µM) in aqueous solution revealed Xe•CB[6] signal with an intensity reduction and peak shift compared to Xe•CB[6] alone. Additionally, a linear change in Xe•CB[6] peak shift was observed with increasing CS DS6 concentration. In order to achieve a similar change in the observed chemical shift of Xe•CB[6] peak, higher concentrations of CS with the lower DS4 are required. This differently pronounced degree of interaction is thus attributed to the presence of different DS available on the CS rims. The specific molecular recognition pattern of CB[6] (5 µM) for SBE chains available on CS (100 µM, CS DS6) was checked by competitively inhibiting the latter by structurally similar butylsulfonate (BS) at different concentrations (100–1000 µM), respectively. The Xe•CB[6] peak of BS-inhibited ternary complexes was noted at −91.09 ppm compared to CB[6] plus CS DS6 (−92.67 ppm) and CB[6] alone (−95.66 ppm), respectively. Our investigations reveal that indeed the SBE chains on CS are essential for facilitating ternary complexation with CB[6] and ^129^Xe probes such spontaneous self-assembly at relatively low concentration (µM regime). Such ^129^Xe HyperCEST-based studies might pave way for studying multiple interactions occurring within complex supramolecular structures, e.g., capsules, machines, necklaces etc. at ease [201].

### 5.5. Functionalized Supramolecular Assemblies

Introduction of a functionalizable handle on the CB[*n*]s structure can lead to the generation of hosts with intriguing properties for different applications such as molecular imaging or targeted drug delivery. Due to high water solubility, mono-functionalization of CB[7] is preferred over modification of the “even” members of the CB[*n*] family]. However, it is a challenging task to introduce functional groups on CB[*n*] skeletons [93,202]. Even more difficult is the synthesis of a mono-functionalized version of CB[7] [94,203]. In this context, Isaacs and coworkers managed to synthesize a mono-functional CB[7] in five steps by utilizing a glycoluril hexamer precursor prior to its reaction with a suitable functionalized glycoluril unit [204,205,206,207]. Similarly, a mono-functionalized CB[6], *i.e.,* monohydroxylated CB[6] [208] can be synthesized by using a modified method involving persulfate salts [209] and/or monohydroxy CB[7] in one step by optimizing the synthesis conditions, respectively. However, the reaction conversions are not quantitative and it requires non trivial and time consuming purification steps to attain monofunctional CB[*n*]s [210]. Despite such tedious synthesis and purification, the as-generated monofunctionalized CB[*n*] finds interesting applications in supramolecular chemistry and drug delivery [207,211]. Investigations of drug-loaded, functionalized CB[*n*]s with HyperCEST can thus provide insights into the cargo stability and release. 

Recently, the first study with a mono-substituted CB[*n*] has investigated the option to detect a specific protein target in solution using ^129^Xe HyperCEST. A biotinylated CB[7] (btCB[7]) was utilized for this work. Originally, mono-functionalized btCB[7] was synthesized as a container molecule for delivering anti-cancer drugs to murine lymphocytic leukemia cancer cells expressing higher levels of biotin receptors [207]. The ^129^Xe HyperCEST of control CB[7], i.e., unmodified CB[7], indicated a response in agreement with previous reports [138]. The Xe•btCB[7] response appeared similar to unmodified CB[7] at δ = −68 ppm albeit with significantly weaker amplitude than for the control CB[7]. The line broadening for saturating dissolved Xe (around 0 ppm) is larger than the anticipated line width derived from the width and intensity of the bound Xe CEST peak. The peak at −68 ppm was thus hypothesized to arise in fact from a low population (~1%) of the stereoisomer *i*CB[7] [70]. The *i*CB[7] possesses a reduced internal cavity diameter (~5.5 Å) due to the inverted flip of a glycoluril unit in its structure. It was hypothesized that a signal from CB[7]-bound Xe is presumably too broad for well resolved CEST detection. However, the existence of Xe•CB[7] manifests in the dissolved Xe peak with broad saturation response [70]. The Xe•*i*btCB[7] response is significantly weaker than the Xe•*i*CB[7], possibly due to less *i*btCB[7] formed during synthesis compared to *i*CB[7]. One might initially not exclude other inter- and intra-molecular interactions that might have played a role in observing a reduced response for Xe•*i*btCB[7], e.g., exclusion complexation between *i*btCB[7] and its own biotin tail or with that of a neighboring *i*btCB[7]. However, the biotinylated tail interaction with CB[7] portals or the cavity has been excluded through MD simulations-based results. Another option might be that *i*btCB[7] dimerizes in solution. Indeed, dimer formation was supported through MALDI and MD simulation-based results. However, the strong avidin binding to biotin is sufficient enough to hamper the btCB[7] dimerization [212] anticipated for such monofunctionalized CB[*n*]. 

The btCB[7] (50 µM) treated with avidin indicated four distinct signals, namely −68 ppm (Xe•*i*btCB[7]), 0 ppm (free Xe) and additional new signals at δ = −40 and 100 ppm, respectively. The resonance at 100 ppm was ascribed to a direct interaction of Xe atoms with avidin. The significant response at δ = −40 ppm was attributed to Xe inside btCB[7] bound to avidin (Xe•btCB[7]-avidin). This was proven by observing no signal in the case of unmodified CB[7] in the presence of avidin. The same signal was also missing for btCB[7] in the presence of avidin that was pre-saturated with biotin. Such interaction between btCB[7] and avidin was estimated to generate ~50% CEST difference adequate for achieving a significant image contrast. Thus, introducing these hosts in close proximity to protein targets generates functionalized CB[*n*]-based biosensors for different imaging applications [139].

## 6. Conclusions and Future Directions

Hyperpolarized noble gases belong to the longest standing applications of sensitivity-enhanced NMR. The good solubility of ^129^Xe in aqueous solutions together with its affinity for binding sites and cavities makes this isotope a rather versatile probe for interrogating supramolecular systems. The combination with the HyperCEST technique provides unprecedented sensitivity, an aspect that is particularly important for CB[6] with low solubility. Concepts like the displacement assays that had originally been implemented with switchable dyes could be transferred to ^129^Xe NMR because of the large chemical shift range and the resulting high selectivity for saturation transfer that brings a switchable NMR signal for transiently bound Xe. 

Initial studies focused on the characterization of host-guest systems and the exchange dynamics for Xe with and without competing guests. Today, the interest goes beyond this physical chemistry aspect as different concepts for versatile biosensor platforms have been presented. CB[*n*]s are an active field of research because of their potential for biomedical applications such as drug delivery [106,207]. Naturally, there is interest in monitoring such systems in vivo where optical detection might be challenging or impractical. CB[6] has also been proposed as a platform for Xe biosensors because of the faster exchange compared to CrA that has been the original host for implementing “functionalized Xe” [142]. A first study demonstrated its use for HyperCEST detection in a preclinical model [213], albeit the full in vitro potential could not yet be translated and the used concentrations (mM range) are not favourable compared to any conventional Gd-based MRI reporters. This is mainly due to competitive binding of other guests that occurs in tissue. The lessons learned from reversible binding of Xe in CB[*n*] supramolecular systems pinpoint critical aspects of a revised design that will also work in living tissue. Tailored cavity sizes and smart rotaxane compounds provide initial clues for future studies. Other possible limitations that might hinder the usage of above CB[*n*]s for ^129^Xe NMR/MRI arise from a lack of a functionalizable handle on the hosts. Installing such moieties might enhance CB[*n*]s solubility and likewise promote further functionalization with appropriate building blocks, e.g., fluorophores, binding units etc. 

Regarding the biocompatibility of such systems for biomedical applications, some cell culture and *in vivo*-based test results are available for CB[*n*] (*n* = 7,8) [214]. They indicate that CB[8] shows no cytotoxicity effects within its solubility range. For CB[7], an *IC*_50_ value of 0.53 mM was determined. In vivo studies revealed a maximum tolerated dosage of 250 mg kg^−1^ for CB[7] in mice. The toxicology has also been investigated in [215] for five members of the CB[*n*] family (*n* = 5–8, 10) with results confirming a very low toxicity of these nanocontainers. Cellular uptake was followed by intracellular release of container-loaded drugs. Altogether, these toxicity profiles encourage further studies with CB[7] as the one with preferable water solubility for medicinal and pharmaceutical use. Xe itself is a harmless noble gas. It passes the blood brain barrier and easily penetrates into cells. The only potential side effect comes from its anesthetic impact [216,217] but it also has a neuroprotective effect [218]. Overall, it has been long approved for diagnostic lung imaging [219] where it provides information on ventilation, parenchymal gas exchange and uptake into red blood cells [220]. The appealing aspect of HyperCEST NMR is that it generates a switchable contrast. Moreover, the frequency selective saturation of Xe is also possible at lower (clinical) field strengths, thus the translation potential from analytical NMR setups to clinical diagnostics is at hand. The scenario in living tissue will presumably rely on constant Xe delivery through the bloodstream after inhalation. The feasibility for such conditions, including macromolecular Xe hosts with relatively fast exchange, has been demonstrated with a pharmacokinetic model for brain tissue [221]. Further translation of these concepts also benefits from recent advancements regarding Xe MRI beyond the gas phase: Detection in other organs like the human brain [222,223,224] as well as the kidneys [225] has become achievable. The potential of such supramolecular systems for diagnostic purposes is evident and together with the drug delivery strategies that are pursued for CB[*n*]s and related compounds, Xe NMR/MRI could make an important contribution to comprehensive therapeutic and diagnostic approaches.
